# Bullying Behaviors in Children and Adolescents: “An Ongoing Story”

**DOI:** 10.3389/fpubh.2014.00007

**Published:** 2014-02-10

**Authors:** Artemis Kimon Tsitsika, Efi Barlou, Elisabeth Andrie, Christine Dimitropoulou, Eleni C. Tzavela, Mari Janikian, Marisa Tsolia

**Affiliations:** ^1^Adolescent Health Unit, “P & A Kyriakou” Children’s Hospital, National and Kapodistrian University of Athens School of Medicine, Athens, Greece; ^2^Second Department of Pediatrics, “P & A Kyriakou” Children’s Hospital, National and Kapodistrian University of Athens School of Medicine, Athens, Greece

**Keywords:** bullying, children, adolescents, intervention

## Abstract

Bullying in school-aged children is a universal problem, which continues to be a serious threat to physical and emotional health of children and adolescents. This article highlights the prevalence, the common characteristics of bullies and victims, as well as the short- and long-term impact of bullying involvement. Key areas highlighted include the efficacy of bullying prevention programs, which can help health care providers to assess and provide interventions to children and adolescents affected by bullying.

## Introduction

Incidents of violence and aggression in schools is a common and expanding phenomenon, which has attracted the interest of scientists, educators, and policy makers for more than three decades in the majority of European countries, North America, and Australia (1). Bullying is defined as negative physical, verbal, or relational actions that (a) have hostile intent, (b) cause distress to the victim, (c) are repeated and (d) involve a power imbalance between perpetrators and victims (2). This definition underlines the key elements that differentiate bullying from other common expressions of aggression among peers, such as fighting, where the imbalance of power is irrelevant, as well as from playful acts based on friendly motives that are part of normal patterns of socialization among youths. Bullying may take multiple forms varying from physical confrontation, teasing, and humiliation to more indirect ways of victimization such as spread of rumors or exclusion from the peer group and social marginalization of the victim (3). During the last years, the significant role of new technologies in youth’s life has contributed to the emergence of cyber bullying as a prevalent phenomenon.

In order to review the epidemiology, identification, and management of bullying and victimization among school-aged children, we conducted a combined computerized and manual systematic database search of medical literature. The respective publications were retrieved from electronic search engines (Medline, PsycINFO, Embase, Scopus, Google Scholar, Ovid, and the Cochrane Library) from 2000 to 2013 using the key words “bullying, children, adolescents, and intervention.” Reference lists were thereafter systematically searched for relevant articles. Eligible studies included studies examining the prevalence, the common characteristics of bullies and victims, as well as the short- and long-term impact of bullying involvement.

## Prevalence

Estimated rates of bullying incidents vary significantly among countries as well as among gender and age groups. For example, the percentage of children who reported involvement in bullying was 13% in the USA (4), 24% in England, and 8% in Germany (5). Evidence from a recent international study indicates that bullying prevalence vary from 32% (among Lithuanian 11-year-old boys) to 2% (among Armenian girls of all ages).

Bullying has been found to vary by age group, which is consistent across the majority of countries. This is mainly due to the fact that the prevalence of bulling declines with age. Many studies suggest that boys are more likely to become perpetrators and/or victims in physical verbal and overall direct forms of bulling, whereas girls are more likely to get involved in indirect forms of bullying. However, further investigation is needed to determine specific gender patterns of bullying behavior among children and adolescents. Previous research indicates also that children of low social–economic status are more vulnerable to bullying (6), whereas other studies do not provide such evidence (7).

## Impact

Bullying is a worrying issue concerning its impact on youth’s health and psychosocial adjustment. Research indicates three groups of individuals who are directly involved in bullying, namely bullies, victims, and bully/victims, each with different, yet sometimes overlapped, characteristics associated to bullying incidents in their lives. The most common negative outcomes associated to victimization are depression (with 29.5% of bullied adolescents reporting depressive symptoms compared to 7.3% of their non-involved peers), anxiety (with more than 50% of victims reporting severe anxiety), and even suicidal ideation. A longitudinal study held by Ttofi et al. (8) provided evidence about the correlation of victimization in adolescence with depressive symptoms in adulthood mostly among females. It is also suggested that victims of bullying manifest high levels of post-traumatic stress (9). Evidence from cross national study of a total of 113,200 students from 25 countries at average age 11.5, 13.5, and 15.5 years suggests the prevalence of externalizing symptoms such as aggressive behavior and conduct problems (10, 11). More precisely in a study survey of 428 undergraduate students based on the National College Health Risk Behavior Survey about alcohol, drug and tobacco, violence and aggression, the Beck Depression Inventory II, and items adapted from the Overt Aggression Scale, evidence showed that almost one third of the sample reported cigarette smoking, 22% moderate depression, 81% drink alcohol, with 58% drinking more than five drinks at least once in the last month. Reports of verbal and physical aggression were also common. Moderate depression was related to cigarette smoking, physical, and verbal aggression, but not to heavy alcohol use (12). In another study of a representative sample of 15,686 students in grades 6 through 10 in public and private schools throughout the United States who completed the World Health Organization’s Health Behaviour in School-aged Children, a correlation was found between bullying and weapon carrying (4). A recently published review of longitudinal studies (1) showed that the probability of being engaged in criminal offending later in life was significantly predicted by bullying perpetration in school. Bullies maladjustment is significant within schooling where they are found to achieve poorer academic records (12, 13).

Most empirical studies show that among youth involved in bullying, the group with the most eminent difficulties in psychological, interpersonal, and academic sectors is that of victims/bullies (14, 15). Their poor functioning indicates them as an especially high-risk group. Although they represent a small group, approximately 6% of adolescents (10), they show high levels of both internalizing and externalizing symptoms in combination with high rates of physical problems possibly related to the accumulative impact of repeated stressful incidents of bullying (11). This subgroup of victims, in fact, reacts aggressively to abuse and has a distinct pattern of maladjustment encompassing both the antisocial tendencies of perpetrators and the emotional problems faced by victims. This leads to long-term mental health and social problems (16).

The largest cross-sectional study provides conclusions about the direction of the observed associations between bullying and facets of psychosocial functioning (17). Despite this limitation, it is largely accepted that bullying should be regarded as a stressful life event contributing to behavior and school adjustment problems (3).

In addition to the short and long-term effects of bullying, there is a connection between the level of bully/victim problems in a classroom or school and aspects of the social climate of the unit concerned. In classroom with high levels of bullying problems, students tend to feel less safe and less satisfied with their school life.

## Causes–Protective Factors

Given the fact that bullying is expressed in setting, it is important to focus on factors that enhance or limit its prevalence and impact. Most studies address bullying as a multifactorial phenomenon influenced by factors associated with the individual, the family, the school, and the broader community levels. Table 1 depicts the most common factors indicated by research as risk or protective ones concerning the emergence of bullying incidence (3, 18–23).

**Table 1:** **Risk and protective factors for bullying in multiple levels**.

	Risk factors	Protective factors
Individual	Poor self-concept	High self-esteem
	Physical disabilities	High social skills levels
	Poor social skills	
	Early aggressiveness	
Family	Authoritarian discipline	Quality communication with parents
	Lack of parental supervision	Parental involvement in school life
	Incidents of domestic violence	
School	Overcrowded schools	Orientation toward learning
	Conflictual school climate	Positive peer role-models
	Violent-tolerant peers	
Neighborhood	Safety concerns	Promotion of sports and recreational facilities

## Basic Principles of Prevention Programs

The identification of multiple risk and protective factors should be considered as a guideline for the development of effective anti-bullying programs. In fact, several programs have been implemented mostly in Northern Europe [e.g., The Olweus Bullying Prevention Program (OBPP) in Norway and Sweden and the KiVa Anti-bullying program in Finland] whose efficacy is demonstrated by empirical evidence (8, 24, 25). The OBPP is used at the school, classroom, and individual levels and includes methods to reach out to parents and the community for involvement and support. School administrators, teachers, and other staff are primarily responsible for introducing and implementing the program. The goals of the program are to reduce existing bullying problems among students, to prevent the development of new bullying problems, and to achieve better peer relations at school. These goals are met through restructuring of the child’s social environment at school. The restructuring is intended to reduce both opportunities and rewards for engaging in bullying behavior and to build a sense of community among students and adults within the school environment. OBPP is designed for students in elementary, middle, and junior high schools (students ages 5–15 years old). OBPP has been more thoroughly evaluated than any other bullying prevention/reduction program so far. Six large-scale evaluations involving more than 40,000 students have documented results such as average reductions of 20–70% in student reports of being bullied and bullying others. Peer and teacher ratings of bullying problems have yielded roughly similar results and clear improvements in the classroom social climate, as reflected in students’ reports of improved order and discipline, more positive social relationships, and more positive attitudes toward schoolwork and school.

KiVa is a research-based anti-bullying program that has been developed in the University of Turku, Finland. The program involves both universal and indicated actions to prevent bullying and to tackle cases of bullying coming to attention. The universal actions are targeted at all students in a school. They refer to efforts made to influence the group norms and to build capacity in all children to behave in constructive ways, to take responsibility for not encouraging bullying, and to support the victims. KiVa has been evaluated in a large randomized controlled trial including 117 intervention schools and 117 control schools. The program has been shown to reduce both self- and peer-reported bullying and victimization significantly. It influences multiple form of victimization, including verbal, physical, and cyberbullying. In addition, positive effects on school liking, academic motivation, and achievement have been reported. KiVa also reduces anxiety and depression and has a positive impact on students’ perception of their peer climate. Finnish data from more than 1000 schools that started the implementation of KiVa in fall 2009 showed that after the first year of implementation, both victimization and bullying had reduced significantly.

Despite the variety of specific goals and methods incorporated in each program, research provides evidence about common core elements that result to the reduction of bullying incidents in schools. The main factors promoting the effectiveness of anti-bullying programs are (1) whole school, non-stigmatizing implementation, (2) involvement of parents in parent training, (3) development of school conferences, (4) quality classroom management–teacher training, (5) duration and intensity of implementation, (6) complexity-number of program components, developmental appropriateness, (7) staff commitment to implement the intervention, and (8) cultural appropriateness.

Despite the specific focus of each study, empirical evidence shows the multiple risks and dangers associated with bullying. It is quite obvious, both by its prevalence rates and its adjustment and function correlates that bullying is one of the major threats for children’s and adolescents’ positive development. Its impact on all facets of youth’s lives leads to the conclusion that it should be considered as a significant international public health issue (26). Furthermore, it is somehow mentioned in most studies that bullying is strongly setting-related, concerning both its form and its prevalence (27). It is of essential importance for further research to focus on the interplay between social parameters and the evolution of phenomena such as bullying especially during periods of transitions such as economic crisis, which is the case for an increasing number of southern and central European countries. Despite the differences observed among countries, research provides evidence that there is a high level of cross-national consistency in the relationship between bullying behaviors and psychosocial adjustment (4).

## Conclusion

Both the content of the impact of bullying on adolescents’ well-being and the pathways through which it is expressed seem to be quite common among countries, a fact that permits the development of prevention and intervention anti-bullying programs with common core elements and goals in a European level. Finally, it is important to mention that given the fact that bullying is such a prevalent phenomenon, future efforts should focus not only on its reduction but also on the fostering of resilience despite its existence.

## Conflict of Interest Statement

The authors declare that the research was conducted in the absence of any commercial or financial relationships that could be construed as a potential conflict of interest.

## References

[1] Ttofi MM Farrington DP Effectiveness of school-based programs to reduce bullying: a systematic and meta-analytic review. J Exp Criminol (2011) 7:27–56 10.1007/s11292-010-9109-1

[2] Olweus D Bullying at School: What We Know and What We Can Do. Malden, MA: Wiley-Blackwell (1993).

[3] Arseneault L Bowes L Shakoor S Bullying victimization in youths and mental health problems: ‘much ado about nothing’. Psychol Med (2010) 40:717–29 10.1017/S0033291709991383 19785920

[4] Nansel TR Overpeck M Pilla RS Ruan WJ Simons-Morton B Scheidt P Bullying behaviors among US youth. JAMA (2001) 285:2094–100 10.1001/jama.285.16.2094 11311098 PMC2435211

[5] Wolke D Woods S Bloomfield L Karstadt L Bullying involvement in primary school and common health problems. Arch Dis Child (2001) 85:197–201 10.1136/adc.85.3.197 11517098 PMC1718894

[6] Reulbach U Ladewig EL Nixon E O’Moore M Williams J O’Dowd T Weight, body image and bullying in 9-year-old children. J Paediatr Child Health (2013) 49:288–93 10.1111/jpc.12159 23530984

[7] Lind J Maxwell G Children’s Experience of Violence at School. Wellington: Office of the Commissioner for Children (1996).

[8] Ttofi MM Farrington DP Lösel F Loeber R The predictive efficiency of school bullying versus later offending: a systematic/meta-analytic review of longitudinal studies. Crim Behav Ment Health (2011) 21:80–9 10.1002/cbm.808 21370293

[9] Schiltz L Houbre B Multidimensional homogeneity analysis with small samples: comparison of different subgroups of people suffering from exclusion. Bull Soc Sci Med Grand Duche Luxemb (2006) 311–25 17124805

[10] Nansel TR Craig W Overpeck MD Saluja G Ruan W Cross-national consistency in the relationship between bullying behaviors and psychosocial adjustment. Arch Pediatr Adolesc Med (2004) 158:730 10.1001/archpedi.158.8.730 15289243 PMC2556236

[11] Wolke D Copeland WE Angold A Costello EJ Impact of bullying in childhood on adult health, wealth, crime, and social outcomes. Psychol Sci (2013) 24:1958–70 10.1177/0956797613481608 23959952 PMC4090076

[12] Roberts SJ Glod CA Kim R Hounchell J Relationships between aggression, depression, and alcohol, tobacco: implications for healthcare providers in student health. J Am Acad Nurse Pract (2010) 22:369–75 10.1111/j.1745-7599.2010.00521.x 20590959

[13] Natvig GK Albrektsen G Qvarnstrøm U Psychosomatic symptoms among victims of school bullying. J Health Psychol (2001) 6:365–77 10.1177/135910530100600401 22049386

[14] Gasteiger-Klicpera B Klicpera C Aggressiveness and social status in the class social structure. Z Kinder Jugendpsychiatr Psychother (1997) 25:139 9459703

[15] Sutton J Smith PK Swettenham J Social cognition and bullying: social inadequacy or skilled manipulation? Br J Dev Psychol (1999) 17:435–50 10.1348/026151099165384

[16] Olweus D Bullying at school: basic facts and effects of a school based intervention program. J Child Psychol Psychiatry (1994) 35:1171–90 10.1111/j.1469-7610.1994.tb01229.x 7806605

[17] Houbre B Tarquinio C Thuillier I Hergott E Bullying among students and its consequences on health. Eur J Psychol Educ (2006) 21:183–208 10.1007/BF03173576

[18] Brendgen M Boivin M Vitaro F Girard A Dionne G Pérusse D Gene-environment interaction between peer victimization and child aggression. Dev Psychopathol (2008) 20:455–71 10.1017/S0954579408000229 18423089

[19] Bowes L Arseneault L Maughan B Taylor A Caspi A Moffitt TE School, neighborhood, and family factors are associated with children’s bullying involvement: a nationally representative longitudinal study. J Am Acad Child Adolesc Psychiatry (2009) 48(5):545–53 10.1097/CHI.0b013e31819cb017 19325496 PMC4231780

[20] Loeber R Hay D Key issues in the development of aggression and violence from childhood to early adulthood. Annu Rev Psychol (1997) 48(1):371–410 10.1146/annurev.psych.48.1.371 9046564

[21] Salmivalli C Bullying and the peer group: a review. Aggress Violent Behav (2010) 15:112–20 10.1016/j.avb.2009.08.007

[22] Espelage DL Swearer SM, editors. Bullying in American Schools: A Social-Ecological Perspective on Prevention and Intervention. Mahwah: Lawrence Erlbaum Associates Publishers (2004).

[23] Pepler DJ Craig WM Connolly JA Yuile A McMaster L Jiang D A developmental perspective on bullying. Aggress Behav (2006) 32:376–84 10.1002/ab.20136

[24] Salmivalli C Poskiparta E Making bullying prevention a priority in Finnish schools: the KiVa antibullying program. New Dir Youth Dev (2012) 2012:41–53 10.1002/yd.20006 22504790

[25] Vreeman RC Carroll AE A systematic review of school-based interventions to prevent bullying. Arch Pediatr Adolesc Med (2007) 161:78–88 10.1001/archpedi.161.1.78 17199071

[26] Gini G Pozzoli T Association between bullying and psychosomatic problems: a meta-analysis. Pediatrics (2009) 123:1059–65 10.1542/peds.2008-1215 19255040

[27] Espelage DL Bosworth K Simon TR Examining the social context of bullying behaviors in early adolescence. J Couns Dev (2000) 78:326–33 10.1016/j.chiabu.2012.12.004 23332296

